# Integrated Multi-Omics Analysis Unveils Distinct Molecular Subtypes and a Robust Immune–Metabolic Prognostic Model in Clear Cell Renal Cell Carcinoma

**DOI:** 10.3390/ijms26073125

**Published:** 2025-03-28

**Authors:** Yilin Zhu, Shihui Yu, Dan Yang, Tian Yu, Yi Liu, Wenlong Du

**Affiliations:** 1 Department of Bioinformatics, School of Life Sciences, Xuzhou Medical University, Xuzhou 221004, China; 2 Department of Biophysics, School of Life Sciences, Xuzhou Medical University, Xuzhou 221004, China; 3 Jiangsu Key Laboratory of New Drug Research and Clinical Pharmacy, Xuzhou Medical University, Xuzhou 221004, China

**Keywords:** clear cell renal cell carcinoma, molecular subtypes, tumor microenvironment, prognostic signature, immunotherapy

## Abstract

Clear cell renal cell carcinoma (ccRCC) is characterized by significant clinical and molecular heterogeneity, with immune and metabolic processes playing crucial roles in tumor progression and influencing patient outcomes. This study aims to elucidate the molecular subtypes of ccRCC by employing non-negative matrix factorization (NMF) clustering on differentially expressed genes (DEGs), thereby identifying distinct transcriptional profiles, immune cell infiltration patterns, and subsequent survival outcomes. Utilizing NMF clustering, we identified two molecular subtypes of ccRCC. We developed a prognostic model using LASSO–Cox regression, validated with multiple datasets and quantitative reverse transcription polymerase chain reaction (qRT-PCR), incorporating ten immunity- and metabolism-related genes (IMRGs) for overall survival (OS) prediction. Immune cell infiltration and tumor mutational burden (TMB) analyses were performed to explore differences between high- and low-risk groups, while Gene Set Enrichment Analysis (GSEA) provided insights into relevant biological pathways. The findings revealed that subtype C1, characterized by a “cold” tumor microenvironment, correlates with better prognostic outcomes compared to subtype C2, which exhibits an immunologically active environment and worse survival prospects. High-risk patients demonstrated poorer OS associated with alterations in immune and metabolic pathways. Immune checkpoint analysis indicated the upregulation of CTLA4, LAG3, and LGALS9 in high-risk patients, suggesting potential therapeutic targets. A nomogram integrating IMRG risk scores with clinical factors displayed high predictive accuracy for 1-, 3-, and 5-year OS. These findings provide novel insights into the molecular heterogeneity of ccRCC and emphasize the interconnected roles of immune dysregulation and metabolic alterations in tumor progression. By identifying key prognostic biomarkers and potential therapeutic targets, this study paves the way for innovative strategies aimed at harnessing immune and metabolic pathways for better clinical outcomes in ccRCC patients.

## 1. Introduction

Clear cell renal cell carcinoma (ccRCC) represents the predominant histological subtype of kidney cancer, comprising about 70–80% of renal malignancies [1,2]. Despite significant advancements in diagnostic and therapeutic strategies, ccRCC remains a highly heterogeneous disease with diverse clinical outcomes, ranging from indolent tumors to highly aggressive and metastatic phenotypes [3,4]. Understanding the molecular determinants of this heterogeneity is essential for accurate prognosis prediction and the development of personalized therapeutic approaches.

Molecular profiling has identified that ccRCC exhibits unique genetic, epigenetic, and transcriptional changes, notably frequent von Hippel–Lindau (VHL) gene mutations, hypoxia-inducible factor (HIF) pathway dysregulation, and metabolic reprogramming [5,6]. These alterations contribute to the unique tumor microenvironment (TME) of ccRCC, which is often enriched with immune cells and stromal components, reflecting a complex interplay between tumor biology and host immune responses [7,8]. The prognostic significance of these molecular and immune changes is not well comprehended, highlighting the urgent need for reliable biomarkers and models to categorize patients by risk and inform clinical management.

Recent transcriptomic advancements have identified DEGs and molecular subtypes in ccRCC, offering insights into its biological heterogeneity [4,9]. NMF clustering is an effective method for identifying molecular subtypes from gene expression data, enabling the classification of patients into distinct biological and clinical groups [10,11]. Subtype-specific differences in gene expression profiles, immune cell infiltration, and survival outcomes have been reported, underscoring the importance of integrating molecular and immune features for comprehensive characterization of ccRCC [12,13].

The immune environment of ccRCC is distinct, marked by a high presence of immune cells like CD8^+^ T cells, regulatory T cells (Tregs), and tumor-associated macrophages (TAMs), alongside increased expression of immune checkpoint molecules such as CTLA4, PD-1, and LAG3 [14,15]. In ccRCC, immune cell infiltration is linked to both favorable and poor outcomes, highlighting the dual role of the immune response in anti-tumor immunity and immune evasion [16,17]. The paradoxical characteristics of the TME underscore the necessity for a more comprehensive understanding of immune cell functional states and their interactions with tumor cells.

In addition to immune dysregulation, ccRCC is characterized by profound metabolic reprogramming, including alterations in glucose, lipid, and amino acid metabolism [4,18]. These metabolic changes not only support tumor growth and survival but also influence the immune microenvironment, creating a complex interplay between metabolic and immune pathways [19,20]. Understanding these interactions is critical for identifying novel therapeutic targets and optimizing combination strategies, such as immune checkpoint inhibitors and metabolic therapies.

This study utilized integrative bioinformatics to thoroughly analyze the molecular and immune diversity of ccRCC. Using NMF clustering on DEGs, we identified two molecular subtypes of ccRCC with distinct gene expression profiles, immune cell infiltration, and survival outcomes. Secondly, we constructed a robust prognostic model utilizing immunity- and metabolism-related genes (IMRGs) through LASSO–Cox regression analysis and confirmed its predictive accuracy across various datasets. Third, we investigated the TME and tumor mutational burden (TMB) in high- and low-risk groups, emphasizing the interaction between immune and metabolic pathways in ccRCC progression.

## 2. Results

### 2.1. Molecular Subtypes and Prognostic Implications in ccRCC

**Figure 1:**
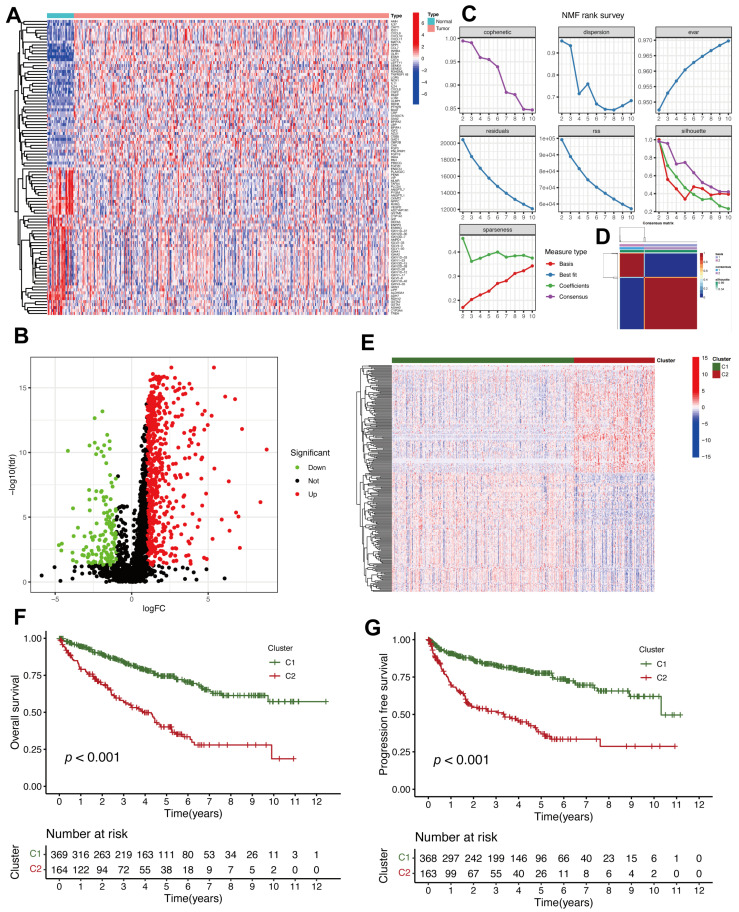
Molecular subgroup characterization and survival analysis in ccRCC. (**A**) Heatmap depicting the top 100 DEGs in ccRCC. (**B**) Volcano plot representing DEG distribution. Selection criteria: |log2FC| > 1 and adjusted *p*-value < 0.05. (**C**) Evaluation parameters for determining optimal NMF clustering number. Parameters include cophenetic correlation, dispersion, evar, residuals, RSS, silhouette width, and sparseness. (**D**) NMF consensus clustering heatmap revealing two distinct subgroups (C1 and C2) within ccRCC samples. (**E**) Expression pattern heatmap demonstrating the two identified clusters (C1 and C2). (**F**) Kaplan–Meier survival curve for OS in C1 and C2. (**G**) Kaplan–Meier survival curve illustrating PFS for clusters C1 and C2.

Differential gene expression analysis was performed using the DESeq2 R package (version 1.40.2) to examine molecular alterations in clear cell renal cell carcinoma (ccRCC) by comparing tumor and normal tissues. Adjusted *p*-values were calculated via the Benjamini–Hochberg method to control the false discovery rate (FDR < 0.05). Figure 1A presents a heatmap illustrating the distinct segregation of tumor and normal samples according to DEGs patterns. The volcano plot (Figure 1B, Supplementary Table S1) illustrates 584 upregulated genes (red) and 141 downregulated genes (green), determined by thresholds of |log2FC| > 1 and an adjusted *p*-value < 0.05. These results indicate significant transcriptional dysregulation in ccRCC, reflecting its distinct molecular characteristics.

NMF clustering was applied to the DEGs to investigate ccRCC heterogeneity. The rank survey metrics (Figure 1C) determined that two clusters, C1 and C2, represent the optimal division of samples. The consensus heatmap (Figure 1D) confirms the stability and robustness of the clustering. Figure 1E presents a heatmap illustrating distinct gene expression profiles between C1 and C2. These profiles suggest that the two clusters may correspond to biologically and clinically distinct subtypes of ccRCC.

Kaplan–Meier survival analysis suggested significant prognostic implications of the molecular subtypes. Figure 1F,G suggest that patients in C1 had significantly improved overall survival (OS, defined as the time from diagnosis to death from any cause) and progression-free survival (PFS, defined as the time from diagnosis to disease progression or death) compared to those in C2 (*p* < 0.001 for both). The molecular subtypes identified through NMF clustering are closely linked to patient outcomes and may act as prognostic indicators.

### 2.2. Tumor Microenvironment Heterogeneity and Immune Characteristics in ccRCC

**Figure 2:**
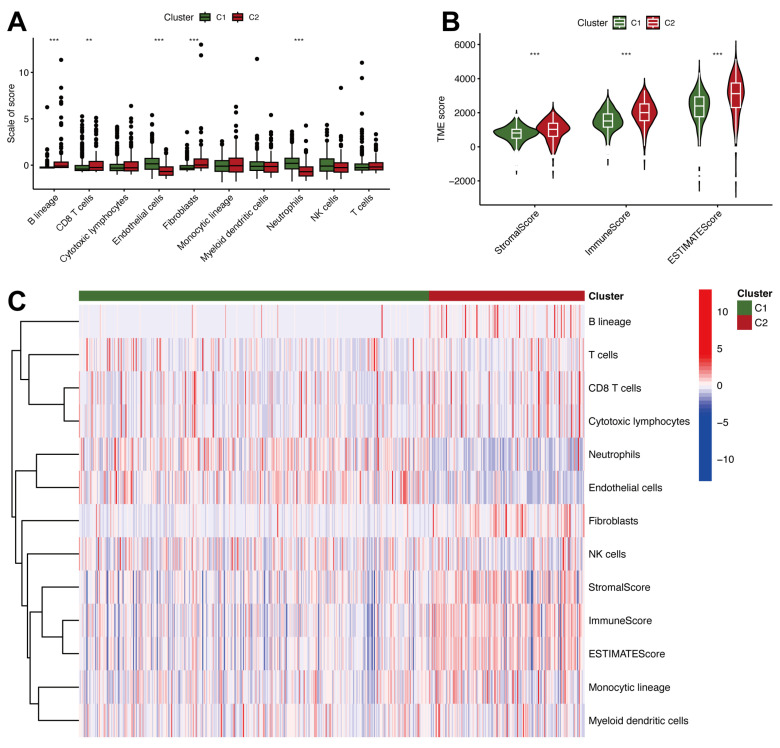
Tumor microenvironment heterogeneity and immune characteristics in ccRCC. (**A**) Boxplots comparing the scores of immune cell infiltration between C1 and C2 in ccRCC. **, *p* < 0.01; ***, *p* < 0.001. (**B**) Violin plots of stromal and immune-related scores, including StromalScore, ImmuneScore, and ESTIMATEScore, for C1 and C2. ***, *p* < 0.001. (**C**) Heatmap of immune cell infiltration and TME-related scores in ccRCC subtypes.

Immune cell infiltration in the two molecular subtypes of ccRCC was evaluated by analyzing the TME. Figure 2A boxplots reveal that C2 had significantly greater immune cell infiltration, including B cells, CD8^+^ T cells, and fibroblasts, than C1 (*p* < 0.01). These findings suggest that C2 is characterized by a more immunologically active tumor microenvironment, likely reflecting an inflammatory phenotype. Violin plots of TME scores (Figure 2B) further highlight the differences between C1 and C2. C2 exhibited significantly elevated StromalScore, ImmuneScore, and ESTIMATEScore compared to C1 (*p* < 0.001), suggesting a greater overall presence of stromal and immune elements in the TME. These elevated TME scores in C2 suggest a complex microenvironment with potential implications for tumor progression and immune evasion mechanisms. A heatmap of immune cell infiltration (Figure 2C) also reveals distinct TME patterns between the two clusters. C2 is characterized by higher expression of immune-related features, whereas C1 shows a relatively immunologically “cold” microenvironment. This distinct immunological landscape also explained that C2 is linked to poor survival (Figure 1F,G).

### 2.3. Creation of a Prognostic Model Utilizing IMRGs in ccRCC

**Figure 3:**
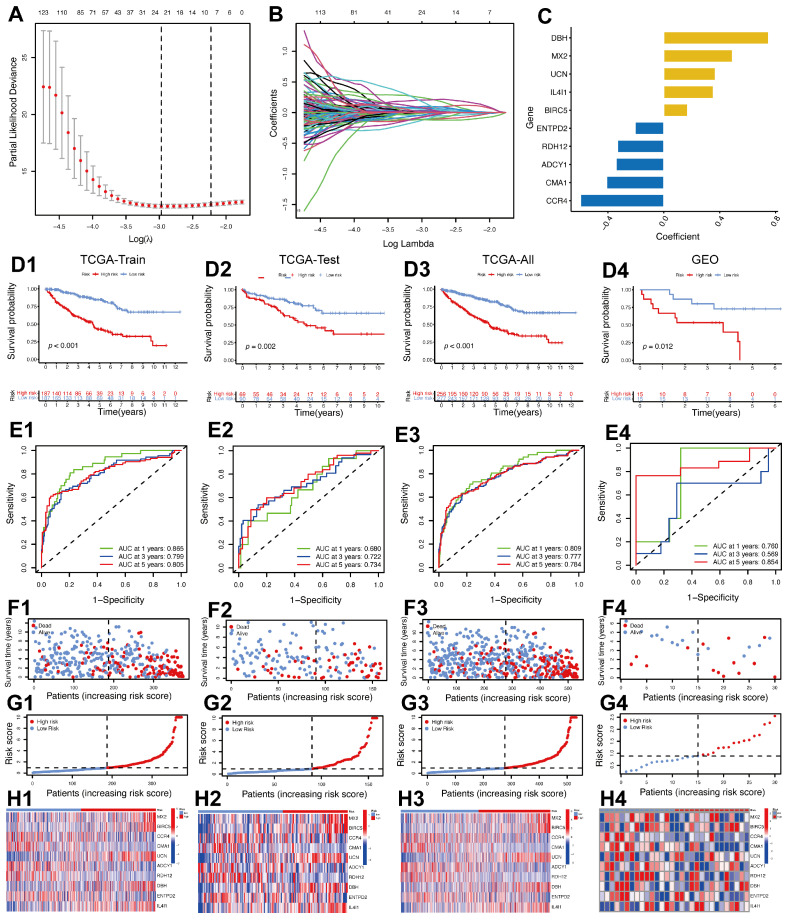
Construction of a prognostic model for ccRCC using genes associated with immunity and metabolism. (**A**) Partial likelihood deviance plot from LASSO–Cox regression analysis showing the optimal lambda value for selecting the 10 IMRGs. (**B**) Coefficient profiles of genes during LASSO–Cox regression analysis. (**C**) Bar plot of gene coefficients in the 10-gene prognostic model. (**D1**–**D4**) Kaplan–Meier survival curves for OS in high-risk and low-risk groups are presented for the TCGA training, TCGA test, TCGA combined, and GEO validation cohorts. (**E1**–**E4**) Time-dependent ROC curves illustrate the 10-gene signature’s predictive performance at 1, 3, and 5 years across the TCGA training, TCGA test, TCGA combined, and GEO validation cohorts. (**F1**–**F4**) Scatterplots illustrate the distribution of survival status (alive or dead) and survival time based on risk scores across the TCGA training, TCGA test, TCGA all, and GEO validation cohorts. (**G1**–**G4**) Distribution plots of risk scores for patients across the TCGA training, TCGA test, TCGA combined, and GEO validation cohorts. Dashed lines distinguish between high-risk and low-risk groups. (**H1**–**H4**) Heatmaps displaying the expression profiles of the 10 IMRGs across the TCGA training, TCGA test, TCGA combined, and GEO validation cohorts.

A prognostic model incorporating 10 IMRGs was constructed using LASSO–Cox regression analysis. The optimal lambda value (lambda.min) was selected based on the minimum partial likelihood deviance (Figure 3A, the left dotted line), balancing predictive accuracy and biological relevance. While lambda.1se provides a more parsimonious model (Figure 3A, the right dotted line), lambda.min was prioritized to retain critical IMRGs for subsequent validation. The coefficients of the selected genes were visualized (Figure 3B). The final model included genes such as DBH, MX2, UCN, IL4I1, BIRC5, ENTPD2, RDH12, ADCY1, CMA1, and CCR4, with their coefficients shown in Figure 3C. These genes represent critical regulators of immune and metabolic processes in ccRCC, highlighting their roles in tumor progression and patient prognosis. Using the 10-gene signature, a risk score was calculated for each patient, classifying them into high-risk and low-risk groups. Kaplan–Meier survival analysis (Figure 3D1–D4) demonstrated that high-risk patients had significantly worse overall survival (OS) compared to low-risk patients in the TCGA training set (*p* < 0.001), TCGA test set (*p* = 0.002), entire TCGA cohort (*p* < 0.001), and the external GEO validation cohort (*p* = 0.012). These findings confirm the model’s prognostic value across various datasets. The model’s predictive accuracy was evaluated using time-dependent Receiver Operating Characteristic (ROC) curves. The AUC values (Figure 3E1–E4) remained relatively high across the training, test, all, and validation cohorts, surpassing 0.7 at 5 years. The findings suggest that the 10-gene signature effectively predicts patient survival. The model’s robustness is further validated by the distribution of risk scores, survival status, and gene expression profiles (Figure 3F1–H4). High-risk patients exhibited worse survival outcomes, as indicated by the survival status plots (Figure 3F1–F4), and distinct expression patterns of the 10 genes, as shown in the heatmaps (Figure 3H1–H4). These findings confirm that the risk score effectively reflects the combined prognostic impact of immunity- and metabolism-related gene expression.

### 2.4. Robust Prognostic Value of the Immunity- and Metabolism-Related Gene Signature Across Clinical Subgroups

**Figure 4:**
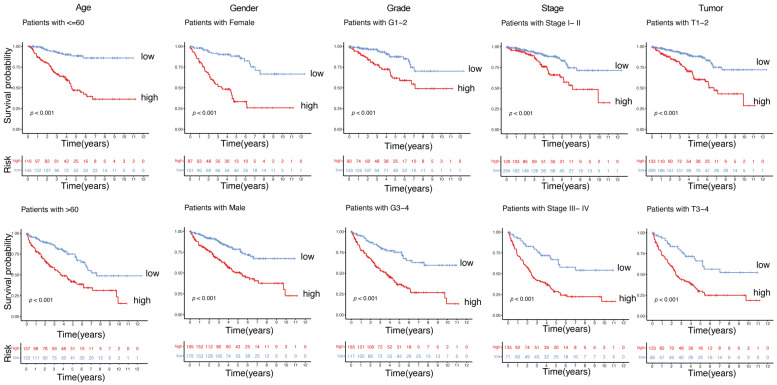
Kaplan–Meier survival curves.

Kaplan–Meier survival analyses were conducted across different clinical subgroups of ccRCC patients to assess the robustness of the gene signature related to immunity and metabolism. The findings indicate that the model retains significant prognostic value across different ages, genders, tumor grades, stages, and sizes (Figure 4, *p* < 0.001). The immunity- and metabolism-related gene signature reliably predicts survival across various patient populations and clinical settings.

### 2.5. Comprehensive Prognostic Evaluation of an Immunity- and Metabolism-Related Gene Model Using Nomogram Analysis

**Figure 5:**
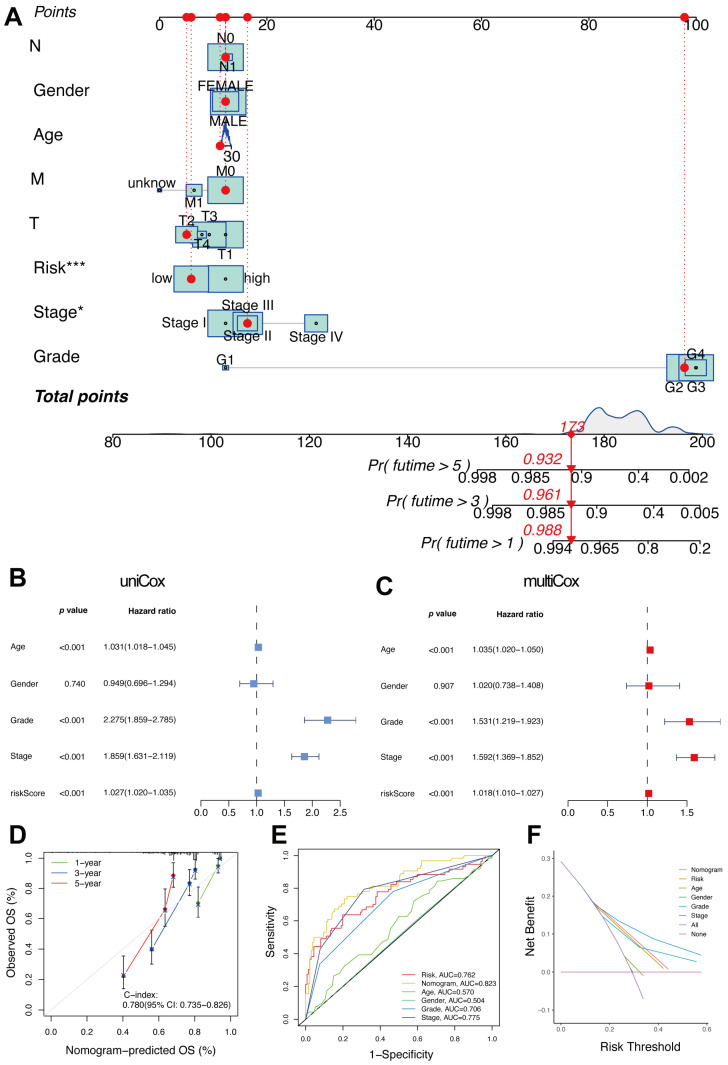
Comprehensive prognostic evaluation of an immunity- and metabolism-related gene model using nomogram analysis. (**A**) Nomogram for predicting 1-, 3-, and 5-year OS in ccRCC, integrating clinical factors (age, gender, grade, stage, T, M, N) with a risk score derived from the immunity- and metabolism-related gene signature. *, *p* < 0.05; ***, *p* < 0.001. (**B**) Forest plot displaying the outcomes of univariate Cox regression analysis. (**C**) Forest plot presenting the results of multivariate Cox regression analysis. (**D**) Calibration plot for the nomogram’s 1-, 3-, and 5-year OS predictions. (**E**) Time-dependent ROC curves comparing the nomogram to individual clinical variables. (**F**) DCA evaluating the clinical utility of the nomogram.

A nomogram incorporating an immunity- and metabolism-related gene signature alongside clinical variables such as age, gender, grade, and stage was developed (Figure 5A). The nomogram provides a score for each variable to predict OS probabilities at 1, 3, and 5 years. The gene signature’s risk score demonstrated the most significant prognostic impact among all variables (*p* < 0.001), underscored by its considerable point contribution. Univariate Cox regression analysis (Figure 5B) revealed that age, tumor grade, tumor stage, and risk score are significant prognostic factors, each with a *p*-value of less than 0.001. Multivariate Cox regression analysis (Figure 5C) confirmed that the risk score (HR = 1.018, *p* < 0.001), along with age (HR = 1.035, *p* < 0.001), tumor grade (HR = 1.531, *p* < 0.001), and tumor stage (HR = 1.592, *p* < 0.001), were independent predictors of OS. The calibration plot (Figure 5D) showed strong concordance between the nomogram-predicted and actual probabilities for 1-, 3-, and 5-year OS. The nomogram demonstrated superior predictive accuracy with a concordance index (C-index) of 0.780 (95% CI: 0.735–0.826). The time-dependent ROC analysis (Figure 5E) confirmed the nomogram’s superior performance with an AUC of 0.823, surpassing individual clinical factors like age (AUC = 0.570), grade (AUC = 0.706), and stage (AUC = 0.775). This suggests that the nomogram enhances the accuracy and comprehensiveness of survival outcome predictions by combining the risk score with clinical features. The clinical utility of the nomogram was assessed using DCA (Figure 5F). The nomogram showed the relatively higher net benefit across a wide range of risk thresholds. This demonstrates the nomogram’s superior ability to guide clinical decision-making and improve personalized risk stratification.

### 2.6. Differential Expression and Prognostic Significance of Model Genes, and Functional Enrichment Analysis of DEGs in High- and Low-Risk Groups

**Figure 6:**
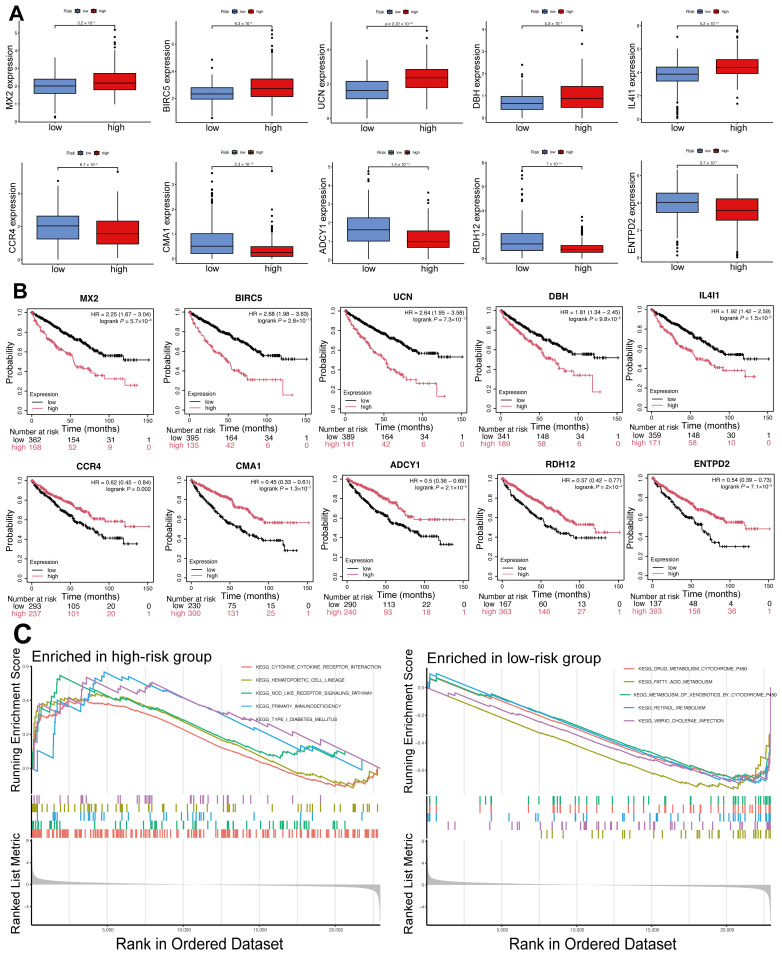
Analysis of model gene expression, prognostic relevance, and functional enrichment of DEGs in high- and low-risk cohorts. (**A**) Boxplots illustrating the expression levels of the ten prognostic model genes in ccRCC, comparing high- and low-risk groups. (**B**) Kaplan–Meier survival curves for the ten prognostic model genes based on the KM Plotter database. (**C**) GSEA outcomes for both high-risk and low-risk cohorts.

The expression levels of the ten genes in the prognostic model were evaluated across high- and low-risk groups. Boxplots (Figure 6A) reveal elevated expression of genes like MX2, BIRC5, UCN, DBH, and IL4I1 in the high-risk group, suggesting their potential oncogenic roles. CCR4, CMA1, ADCY1, RDH12, and ENTPD2 exhibited higher expression levels in the low-risk group, indicating their possible tumor-suppressive functions. The prognostic value of the model genes was further assessed by generating Kaplan–Meier survival curves using the KMplot database (Figure 6B). The findings indicated that elevated levels of MX2, BIRC5, UCN, DBH, and IL4I1 were significantly linked to poorer OS, with Hazard Ratios between 1.81 and 2.68 (*p* < 0.001). In contrast, elevated levels of CCR4, CMA1, ADCY1, RDH12, and ENTPD2 correlated with improved OS, exhibiting hazard ratios between 0.45 and 0.62 (*p* < 0.01). These findings suggest that the identified IMRGs are crucial to the prognostic model and possess independent prognostic value. Their differential roles in promoting or suppressing tumor progression highlight their potential as therapeutic targets or biomarkers in ccRCC.

GSEA was conducted to determine the biological pathways linked to the high- and low-risk groups (Figure 6C). In the high-risk group, the enriched pathways cytokine–cytokine receptor interaction, hematopoietic cell lineage, and NOD-like receptor signaling are associated with immune and inflammatory responses. The findings indicate that an imbalanced immune microenvironment might play a role in the unfavorable prognosis observed in the high-risk group. In the low-risk group, the enriched pathways were mainly linked to metabolic processes, such as drug metabolism via cytochrome P450, fatty acid biosynthesis, and retinol metabolism. The results suggest that improved metabolic functions might offer protection to the low-risk group.

### 2.7. Analysis of Prognostic Gene Expression Differences Between Tumor and Normal Tissues, with Validation in ccRCC Cell Lines

**Figure 7:**
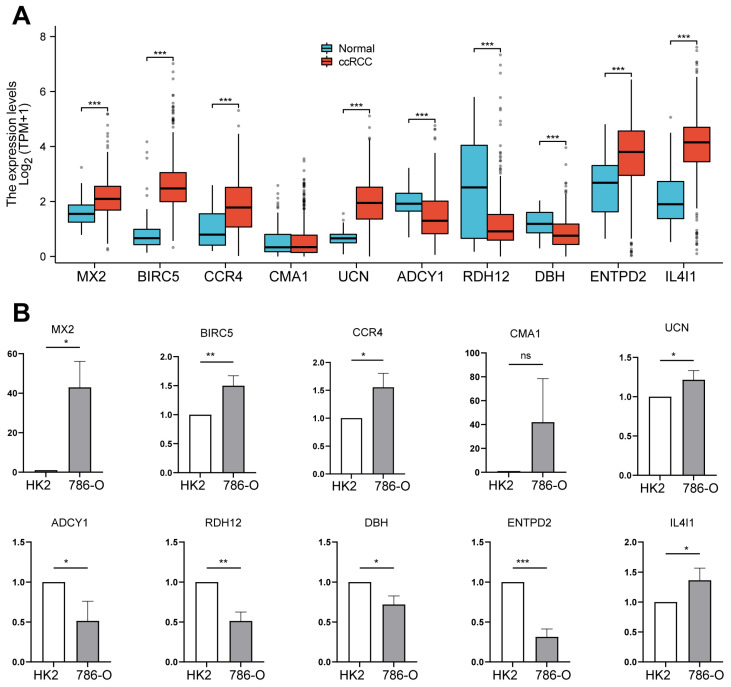
The differential expression of prognostic genes between tumor and normal tissues, with validation conducted in ccRCC cell lines. (**A**) Boxplots showing differential expression of the ten prognostic model genes in ccRCC tissues (red) vs. adjacent normal tissues (blue). *** *p* < 0.001. (**B**) qRT-PCR analysis of the ten prognostic model genes in HK2 (normal renal cells) and 786-O (ccRCC cells). * *p* < 0.05, ** *p* < 0.01, *** *p* < 0.001, and ns for not significant.

The expression levels of the ten prognostic model genes were analyzed in both tumor and adjacent normal tissues of ccRCC samples. Boxplots (Figure 7A) demonstrate that the expression levels of MX2, BIRC5, CCR4, UCN, IL4I1, and ENTPD2 are significantly higher in ccRCC tissues compared to normal tissues (*p* < 0.001). ADCY1, DBH, and RDH12 exhibited significant downregulation in ccRCC tissues compared to adjacent normal tissues (*p* < 0.001). These findings confirm the dysregulation of IMRGs in ccRCC, consistent with their roles (apart from DBH, CCR4, CMA1 and ENTPD2) in tumor progression and their inclusion in the prognostic model. To further validate these findings, qRT-PCR analysis was performed on HK2 (normal renal cells) and 786-O (ccRCC cells) cell lines (Figure 7B). The findings aligned with the tissue expression data, except for ENTPD2 expression.

### 2.8. Immune Cell Infiltration and Tumor Mutational Burden in High- and Low-Risk Groups

**Figure 8:**
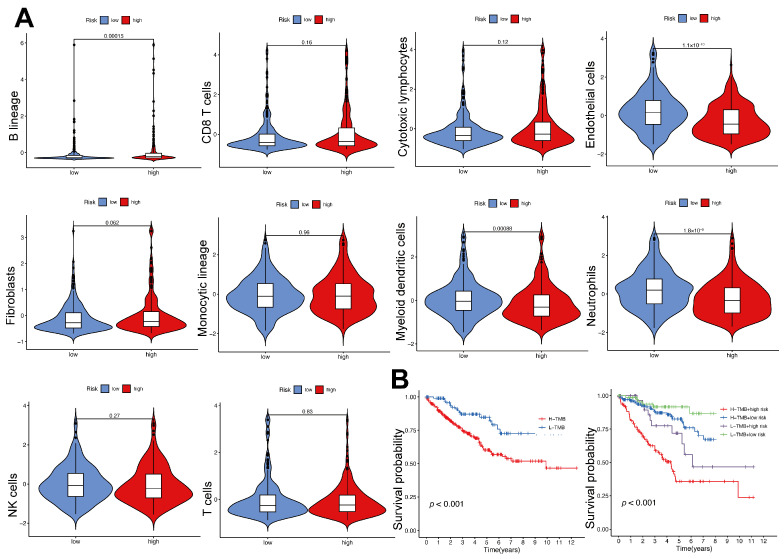
The variations in immune cell infiltration and TMB across high-risk and low-risk groups. (**A**) Violin plots showcase the differences in immune cell infiltration between high- and low-risk groups. (**B**) Kaplan–Meier survival curves illustrate the relationship between TMB and risk categories.

Figure 8A illustrates the analysis of immune cell infiltration patterns to explore the variations between high- and low-risk groups. The findings indicated a significant enrichment of B lineage cells in the high-risk group (*p* < 0.001), implying their potential contribution to tumor progression and adverse prognosis. The low-risk group exhibited a higher abundance of endothelial cells, myeloid dendritic cells, and neutrophils (*p* < 0.001), suggesting a link to improved prognosis. There were no notable differences between the two groups in terms of CD8^+^ T cells, cytotoxic lymphocytes, NK cells, T cells, fibroblasts, or monocytic lineage cells. The results indicate that different immune cell populations within the TME may affect clinical outcomes in patients with varying risk levels. TMB was evaluated to explore its prognostic relevance (Figure 8B). Patients with high TMB had significantly worse survival outcomes compared to those with low TMB (*p* < 0.001). Stratified analysis revealed that patients in the high-risk category with elevated TMB experienced the poorest survival, while those in the low-risk category with lower TMB showed the best survival. These results highlight the prognostic value of combining TMB with the immunity- and metabolism-related risk model.

### 2.9. Expression of Prognostic Model Genes in Immune Cell Subsets in ccRCC

**Figure 9:**
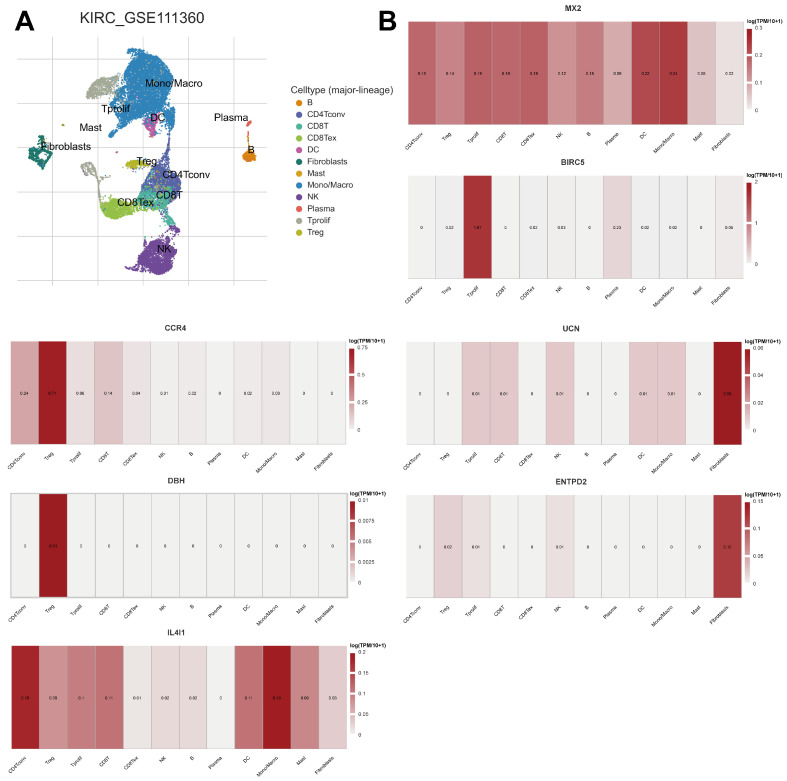
Expression of prognostic model genes in immune cell subsets in ccRCC. (**A**) Uniform Manifold Approximation and Projection (UMAP) plot showing clustering of immune and stromal cell subpopulations in the ccRCC microenvironment based on scRNA-seq data. (**B**) Heatmaps of model gene expression across immune cell subpopulations.

We examined the expression levels of prognostic model genes across different immune cell subsets to clarify their role in the TME (Figure 9A). The findings demonstrated unique expression patterns of the model genes among various immune cell types. MX2 exhibited elevated expression in various immune cell types, notably in dendritic cells and monocytes/macrophages, indicating its extensive role in regulating immune responses. BIRC5 exhibited selective expression, with predominant presence in proliferating T cells (Tprolif), indicating its potential role in T cell proliferation and activation. CCR4 was highly expressed in CD4^+^ conventional T cells (CD4Tconv) and regulatory T cells (Treg), indicating its possible involvement in T cell trafficking, migration, and immune response regulation. IL4I1 showed widespread expression across various immune cell types, with particularly high levels in monocytes/macrophages and CD4Tconv, indicating its potential role in regulating the function and activity of these key immune cell populations. ENTPD2 and UCN displayed more restricted expression patterns, primarily in fibroblasts, suggesting their specialized roles in the tumor microenvironment. DBH showed selective expression in certain immune cell populations, particularly in Treg, indicating its potential role in the differentiation, function, and immunomodulatory activities of these regulatory T cell subsets.

These findings indicate that the prognostic model genes play diverse roles in different immune cell subsets, reflecting their multifaceted functions in the TME.

### 2.10. Immune Checkpoint Gene Expression Varies Between High- and Low-Risk ccRCC Groups

**Figure 10:**
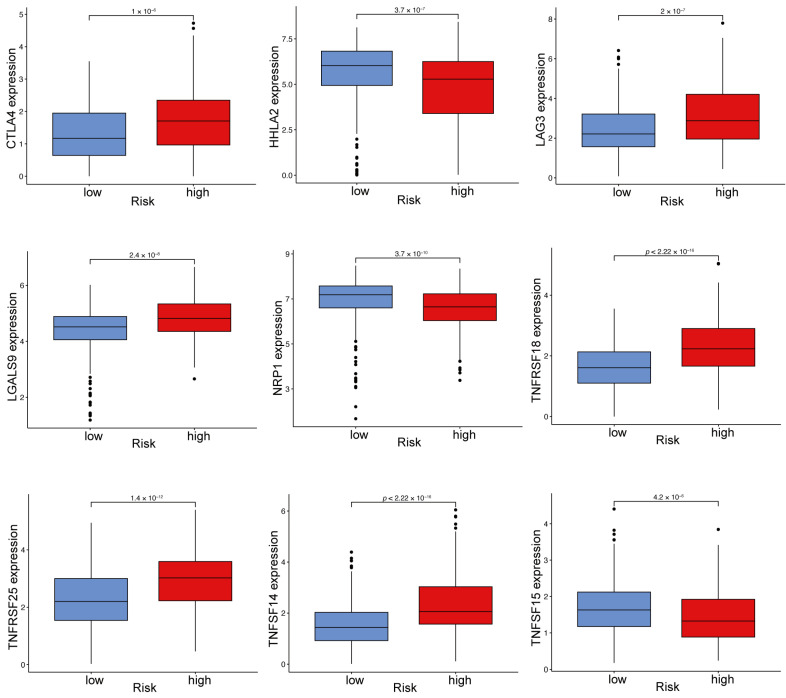
Comparison of immune checkpoint gene expression between high-risk and low-risk groups in ccRCC. Boxplots illustrating the expression levels of immune checkpoint genes in high-risk versus low-risk ccRCC groups. *p* < 1 × 10^−5^ as the screening criterion.

Significant differences in immune checkpoint gene expression were observed between high- and low-risk groups. The findings reveal that immune checkpoint genes such as CTLA4, LAG3, LGALS9, TNFRSF18, TNFRSF25, and TNFSF14 are expressed at significantly elevated levels in the high-risk group relative to the low-risk group (Figure 10). Elevated CTLA4 and LAG3 expression reflects increased immune checkpoint activity, likely contributing to T cell exhaustion and immune suppression in the tumor microenvironment of high-risk patients. These genes are key targets of immune checkpoint inhibitors (ICIs), implicating their therapeutic potential in this group. The upregulation of LGALS9, a ligand for TIM-3, highlights its role in promoting T cell dysfunction and immune evasion [21,22]. This indicates that therapies aimed at LGALS9-mediated immune suppression could be advantageous for the high-risk group. Increased TNFRSF18 and TNFRSF25 expression suggests heightened regulatory T cell (Treg) activity and immune response modulation in high-risk patients [23,24]. These findings underline the immunosuppressive nature of the tumor microenvironment in this group. Elevated TNFSF14 expression indicates its role in influencing the tumor microenvironment and facilitating immune evasion by tumors.

The low-risk group exhibited significantly elevated expression levels of HHLA2, NRP1, and TNFSF15 (Figure 10), suggesting a less immunosuppressive tumor microenvironment. In the low-risk group, increased HHLA2 expression might play a role in sustaining a balanced immune response. This could contribute to better immune surveillance and reduced tumor progression. Increased NRP1 expression is associated with angiogenesis and immune regulation [25], potentially reflecting a more active immune microenvironment in low-risk patients. Elevated TNFSF15 expression indicates increased anti-angiogenic and pro-inflammatory activity, potentially leading to a better prognosis in the low-risk group.

## 3. Discussion

This study offers an in-depth analysis of molecular subtypes, tumor microenvironment, and prognostic factors in ccRCC, revealing new insights into its molecular diversity and potential therapeutic targets. The identification of two molecular subtypes using NMF clustering revealed distinct transcriptional, prognostic, and immune characteristics, highlighting the biological complexity of ccRCC. These findings align with previous reports that ccRCC is a highly heterogeneous disease with unique molecular alterations and immune landscapes influencing patient outcomes [4,26].

Our results demonstrate that C1 exhibits favorable survival outcomes and a less immune-active tumor microenvironment, whereas C2 is associated with poor prognosis and a more immunologically active yet immunosuppressive phenotype. This observation is consistent with prior studies showing that increased immune cell infiltration does not always correlate with better survival. Instead, certain immune-enriched tumor microenvironments may reflect chronic inflammation and immune evasion mechanisms, which are detrimental to prognosis [27].

The development of a 10-gene prognostic model based on IMRGs underscores the critical interplay between immune and metabolic pathways in ccRCC. High-risk patients, characterized by upregulated expression of genes such as MX2, BIRC5, and IL4I1, exhibited worse survival outcomes. These genes are associated with tumor progression and immune modulation in various cancers. BIRC5 (survivin) is a recognized anti-apoptotic protein associated with tumor proliferation and therapy resistance [28]. Similarly, IL4I1 has been shown to suppress T cell activation and promote immune escape in various tumor types [29]. Conversely, low-risk patients demonstrated higher expression of metabolic genes, such as CMA1 and ADCY1, which may enhance metabolic homeostasis and anti-tumor immunity. This is consistent with prior research suggesting that metabolic reprogramming influences tumor progression and therapeutic response in ccRCC [18]. Our results confirm the prognostic importance of these genes and endorse their potential as therapeutic targets.

Integrating TMB with the IMRG-based risk model indicated that elevated TMB correlates with reduced survival, especially among high-risk patients. This observation is in line with studies suggesting that while high TMB may increase neoantigen load and immune recognition, it can also drive immunosuppressive mechanisms that facilitate tumor progression [30]. Integrating TMB with the risk model underscores the significance of including genomic and immune-related characteristics for enhanced prognostic stratification in ccRCC.

The differing immune checkpoint profiles between high- and low-risk groups offer additional understanding of the immunosuppressive mechanisms in ccRCC. High-risk tumors exhibited increased expression of immune checkpoint genes like CTLA4, LAG3, and LGALS9, linked to immune evasion and unfavorable prognosis. These findings are consistent with prior studies demonstrating that increased immune checkpoint activity contributes to T cell dysfunction and tumor immune escape in ccRCC [14]. LGALS9, a TIM-3 ligand, plays a crucial role in immune suppression within the tumor microenvironment, indicating its potential as a therapeutic target for high-risk patients [31]. In contrast, low-risk tumors exhibited higher expression of HHLA2, NRP1 and TNFSF15, which are associated with a more balanced immune response and anti-tumor activity. These results underscore the potential for personalized immunotherapy strategies targeting immune checkpoints based on risk stratification.

The differential expression of the model genes in various immune cell types underscores their critical roles in modulating immune responses in ccRCC. Elevated gene expression in particular immune cell subsets indicates their role in innate and adaptive immune functions. For example, the elevated expression of MX2 and BIRC5 in CD8^+^ T cells indicates their potential to enhance T cell-mediated cytotoxicity, which is crucial for anti-tumor immunity. Furthermore, the unique expression patterns of these genes in immune cells may influence the varying prognoses seen between high- and low-risk groups. The high-risk group, characterized by upregulation of genes like MX2, BIRC5, and UCN, may exhibit a more immunosuppressive microenvironment, leading to poor prognosis. The low-risk group, characterized by elevated expression of genes like CCR4 in CD4+ conventional T cells (CD4Tconv) and regulatory T cells (Treg), likely possesses a more immune-active microenvironment, leading to improved survival outcomes.

The variation in enriched pathways between high- and low-risk groups underscores the molecular diversity of ccRCC. High-risk tumors showed enrichment in immune-related pathways, including cytokine–cytokine receptor interaction and hematopoietic cell lineage, indicating an inflamed yet immunosuppressive microenvironment. In contrast, low-risk tumors showed enrichment in metabolic pathways, including fatty acid biosynthesis and retinol metabolism, which may contribute to a more favorable prognosis. These findings are consistent with previous research suggesting that metabolic adaptability and immune regulation are key determinants of ccRCC progression and therapy response [20,32].

Our study has important clinical implications. The 10-gene risk model and associated nomogram provide a robust tool for patient stratification and prognosis prediction. Moreover, the identification of distinct immune checkpoint profiles and metabolic pathways offers a rationale for stratified therapeutic approaches. High-risk patients may benefit from immune checkpoint blockade therapies targeting CTLA4, LAG3, or TIM-3/LGALS9, while low-risk patients with active metabolic pathways may respond to therapies aimed at enhancing their metabolic functions or targeting angiogenesis. These findings align with the growing emphasis on precision oncology and the need for personalized treatment strategies in ccRCC [7].

Despite its strengths, this study has some limitations. To begin with, the study offers insights into the prognostic and biological roles of the identified genes; however, functional validation experiments are necessary to clarify their specific mechanisms in ccRCC progression. Additionally, while the prognostic model demonstrated robust performance in the training cohort (AUC > 0.7 at 3 and 5 years), lower AUC values in the test set (TCGA: 0.680 at 1 year) and external validation cohort (GEO: 0.569 at 3 years) highlight potential variability across datasets. These discrepancies may reflect differences in patient demographics, treatment protocols, or batch effects, underscoring the need for prospective validation in multi-center cohorts. Finally, the potential impact of treatment modalities on the identified molecular subtypes and risk groups was not explored, warranting further investigation.

## 4. Materials and Methods

### 4.1. Data Acquisition and Preprocessing

Gene expression datasets and clinical information for clear cell renal cell carcinoma (ccRCC) patients were obtained from publicly available repositories, including The Cancer Genome Atlas (TCGA, retrieved on 20 April 2023) and the Gene Expression Omnibus (GEO, dataset GSE29609).

### 4.2. Differential Gene Expression Analysis

The DESeq2 R package (version 1.40.2) [33] was utilized to identify differentially expressed genes (DEGs) between tumor and normal tissues. Genes were considered significantly differentially expressed if they exhibited an absolute log2 fold change exceeding 1 and an adjusted *p*-value of less than 0.05, with corrections for false discovery rate applied using the Benjamini–Hochberg method [34]. Heatmaps and volcano plots were generated to visualize DEG patterns using the ComplexHeatmap (version 2.6.2) and ggplot2 R packages (version 3.3.5) [35,36].

### 4.3. Clustering Using Non-Negative Matrix Factorization (NMF)

Molecular subtypes were identified by applying NMF clustering to the DEGs using the NMF R package (version 0.23.0) [37]. The ideal cluster count was identified using cophenetic correlation, dispersion, silhouette width, and residual sum of squares (RSS). Consensus clustering was used to validate subtype stability [38]. Heatmaps of the identified subtypes were generated, and their distinct transcriptional profiles were analyzed.

### 4.4. Kaplan–Meier Survival Analysis

The prognostic significance of the identified molecular subtypes was evaluated using Kaplan–Meier survival analysis. Overall survival (OS) and progression-free survival (PFS) were compared among subtypes using the survival (version 3.2.10) and survminer R packages (version 0.4.9) [39]. Log-rank tests (two-tailed, α = 0.05) were applied to evaluate statistical significance for Kaplan–Meier survival curves, and hazard ratios (HRs) with 95% confidence intervals (CIs) were calculated using univariate Cox proportional hazards regression. The KM Plotter database (https://kmplot.com/analysis/, accessed on 4 November 2024) was employed to analyze the effect of 10 model gene expressions on patient outcomes.

### 4.5. Tumor Microenvironment and Immune Analysis

The ESTIMATE algorithm (version 1.0.13) was used to evaluate the TME and immune cell infiltration by computing the StromalScore, ImmuneScore, and ESTIMATEScore [40]. Single-sample gene set enrichment analysis (ssGSEA), implemented with the GSVA R package (version 1.38.2) [41], was applied to quantify immune cell infiltration. Boxplots and violin plots were utilized to compare immune and stromal components across subtypes. Heatmaps of immune cell infiltration were created to depict subtype-specific TME characteristics. The Tumor Immune Single Cell Hub (TISCH) database (http://tisch.comp-genomics.org, accessed on 12 November 2024) was used to analyze the expression of prognostic model genes in immune cell subsets within ccRCC.

### 4.6. Construction of the Prognostic Gene Signature

A prognostic model was developed based on genes related to immunity and metabolism. Candidate genes were selected by intersecting differentially expressed genes (DEGs) with immune- and metabolism-related gene sets from the Molecular Signatures Database (MSigDB) (version 7.5.1) [42] (refer to Supplementary Table S2). The glmnet R package (version 4.1.3) was applied to perform LASSO–Cox regression analysis, identifying the most predictive genes [43]. The final model comprised 10 genes, and risk scores for each patient were calculated using a weighted linear combination of gene expression levels and LASSO-derived coefficients.

### 4.7. Model Validation and Performance Evaluation

The prognostic model was validated using the TCGA dataset and an independent GEO cohort. Patients were divided into high-risk and low-risk groups based on the median risk score, and Kaplan–Meier survival curves were generated to evaluate overall survival. The timeROC R package (version 0.4) [44] was employed to measure the model’s predictive accuracy through time-dependent ROC curves at 1, 3, and 5 years. The model’s performance was assessed by calculating the area under the curve (AUC).

### 4.8. Functional Enrichment Analysis

Gene Set Enrichment Analysis (GSEA) was performed to identify biological pathways associated with high-risk and low-risk groups. The GSEA software (version 4.2.3) was utilized along with annotated gene sets from MSigDB, including KEGG and Reactome pathways [45].

### 4.9. Analysis of Immune Checkpoints and TMB

Boxplots were employed to compare the expression levels of immune checkpoint genes between high-risk and low-risk groups, highlighting significant differences (*p* < 1 × 10^−5^ TMB was calculated using somatic mutation data from TCGA and expressed as mutations per megabase [46]. Kaplan–Meier survival analysis was conducted to evaluate the prognostic impact of combining TMB with the risk model.

### 4.10. Nomogram Development and Validation

A nomogram integrating the risk score with clinical factors, including age, gender, tumor grade, and stage, was constructed using the rms R package (version 6.2.0) [47]. Calibration plots were created to evaluate the concordance between predicted and observed survival probabilities at 1, 3, and 5 years. The clinical usefulness of the nomogram was analyzed through decision curve analysis (DCA) [48].

### 4.11. Validation of Prognostic Genes in Cell Lines

The expression levels of the 10 prognostic genes were validated in ccRCC tissues and cell lines. Differential expression between tumor and normal tissues was analyzed using the TCGA-KIRC dataset. Gene expression was further confirmed through quantitative real-time PCR (qRT-PCR) performed on HK2 (normal renal cells) and 786-O (ccRCC cells) (Zhong Qiao Xin Zhou Biotechnology Co., Ltd., Beijing, China). Relative expression levels were calculated using the 2^−ΔΔCt^ method, with GAPDH as the normalization control [49]. The primers used in this study are listed in Supplementary Table S3.

## 5. Conclusions

In conclusion, this study highlights the molecular and immune heterogeneity of ccRCC and provides a comprehensive framework for prognostic stratification and therapeutic targeting. The integration of immune and metabolic features represents a promising approach for improving patient outcomes and advancing precision oncology in ccRCC.

## Data Availability

The datasets used and/or analyzed during the current study are available from the TCGA, GEO, KM Plotter and TISCH databases.

## Supplementary Materials

The supporting information can be downloaded at: https://www.mdpi.com/article/10.3390/ijms26073125/s1.

## Abbreviations

The following abbreviations are used in this manuscript:

ccRCC	Clear cell renal cell carcinoma
TME	Tumor microenvironment
VHL	von Hippel–Lindau
HIF	Hypoxia-inducible factor
TAMs	Tumor-associated macrophages
Tregs	Regulatory T cells
NMF	Non-negative matrix factorization
LASSO	Least Absolute Shrinkage and Selection Operator
DEGs	Differentially expressed genes
qRT-PCR	Quantitative reverse transcription polymerase chain reaction
GSEA	Gene Set Enrichment Analysis
ssGSEA	Single-sample gene set enrichment analysis
OS	Overall survival
PFS	Progression-free survival
TMB	Tumor mutational burden
AUC	Area Under the Curve
ROC	Receiver Operating Characteristic
HR	Hazard ratio
CI	Confidence interval
C-index	Concordance index
TCGA	The Cancer Genome Atlas
GEO	Gene Expression Omnibus
KM	Kaplan–Meier
TISCH	Tumor Immune Single Cell Hub
MSigDB	Molecular Signatures Database
IMRGs	Immunity- and metabolism-related genes
DCA	Decision Curve Analysis
UMAP	Uniform Manifold Approximation and Projection

## References

[1] Ljungberg B., Albiges L., Abu-Ghanem Y., Bedke J., Capitanio U., Dabestani S., Fernández-Pello S., Giles R.H., Hofmann F., Hora M. (2022). European Association of Urology Guidelines on Renal Cell Carcinoma: The 2022 Update. Eur. Urol..

[2] Siegel R.L., Miller K.D., Wagle N.S., Jemal A. (2023). Cancer statistics, 2023. CA Cancer J. Clin..

[3] Jonasch E., Gao J., Rathmell W.K. (2014). Renal cell carcinoma. BMJ.

[4] Linehan W.M., Schmidt L.S., Crooks D.R., Wei D., Srinivasan R., Lang M., Ricketts C.J. (2019). The Metabolic Basis of Kidney Cancer. Cancer Discov..

[5] The Cancer Genome Atlas Research Network (2013). Comprehensive molecular characterization of clear cell renal cell carcinoma. Nature.

[6] Gossage L., Eisen T., Maher E.R. (2015). VHL, the story of a tumour suppressor gene. Nat. Rev. Cancer.

[7] Motzer R.J., Jonasch E., Agarwal N., Alva A., Baine M., Beckermann K., Carlo M.I., Choueiri T.K., Costello B.A., Derweesh I.H. (2022). Kidney Cancer, Version 3.2022, NCCN Clinical Practice Guidelines in Oncology. J. Natl. Compr. Cancer Netw..

[8] Rosellini M., Marchetti A., Mollica V., Rizzo A., Santoni M., Massari F. (2023). Prognostic and predictive biomarkers for immunotherapy in advanced renal cell carcinoma. Nat. Rev. Urol..

[9] Zhang S., Zhang E., Long J., Hu Z., Peng J., Liu L., Tang F., Li L., Ouyang Y., Zeng Z. (2019). Immune infiltration in renal cell carcinoma. Cancer Sci..

[10] Zhang Z., Ji M., Li J., Wu Q., Huang Y., He G., Xu J. (2021). Molecular Classification Based on Prognostic and Cell Cycle-Associated Genes in Patients with Colon Cancer. Front. Oncol..

[11] Huo X., Yang M., Zhang X., Wang S., Sun H. (2022). Identification of Tumor Microenvironment Scoring Scheme Based on Bioinformatics Analysis of Immune Cell Infiltration Pattern of Ovarian Cancer. J. Oncol..

[12] Chakiryan N.H., Hajiran A., Kim Y., Aydin A.M., Zemp L., Katende E., Nguyen J., Fan W., Cheng C.H., Lopez-Blanco N. (2022). Correlating Immune Cell Infiltration Patterns with Recurrent Somatic Mutations in Advanced Clear Cell Renal Cell Carcinoma. Eur. Urol. Focus.

[13] Hua X., Ge S., Zhang J., Xiao H., Tai S., Yang C., Zhang L., Liang C. (2021). A costimulatory molecule-related signature in regard to evaluation of prognosis and immune features for clear cell renal cell carcinoma. Cell Death Discov..

[14] Braun D.A., Hou Y., Bakouny Z., Ficial M., Sant’Angelo M., Forman J., Ross-Macdonald P., Berger A.C., Jegede O.A., Elagina L. (2020). Interplay of somatic alterations and immune infiltration modulates response to PD-1 blockade in advanced clear cell renal cell carcinoma. Nat. Med..

[15] McDermott D.F., Huseni M.A., Atkins M.B., Motzer R.J., Rini B.I., Escudier B., Fong L., Joseph R.W., Pal S.K., Reeves J.A. (2018). Clinical activity and molecular correlates of response to atezolizumab alone or in combination with bevacizumab versus sunitinib in renal cell carcinoma. Nat. Med..

[16] Motzer R.J., Rini B.I., McDermott D.F., Arén Frontera O., Hammers H.J., Carducci M.A., Salman P., Escudier B., Beuselinck B., Amin A. (2019). Nivolumab plus ipilimumab versus sunitinib in first-line treatment for advanced renal cell carcinoma: Extended follow-up of efficacy and safety results from a randomised, controlled, phase 3 trial. Lancet Oncol..

[17] Fridman W.H., Pagès F., Sautès-Fridman C., Galon J. (2012). The immune contexture in human tumours: Impact on clinical outcome. Nat. Rev. Cancer.

[18] Wettersten H.I., Aboud O.A., Lara P.N., Weiss R.H. (2017). Metabolic reprogramming in clear cell renal cell carcinoma. Nat. Rev. Nephrol..

[19] Choueiri T.K., Motzer R.J. (2017). Systemic Therapy for Metastatic Renal-Cell Carcinoma. N. Engl. J. Med..

[20] Hsieh J.J., Purdue M.P., Signoretti S., Swanton C., Albiges L., Schmidinger M., Heng D.Y., Larkin J., Ficarra V. (2017). Renal cell carcinoma. Nat. Rev. Dis. Prim..

[21] Yang R., Hung M.C. (2017). The role of T-cell immunoglobulin mucin-3 and its ligand galectin-9 in antitumor immunity and cancer immunotherapy. Sci. China Life Sci..

[22] Tao J., Li L., Wang Y., Fu R., Wang H., Shao Z. (2016). Increased TIM3^+^CD8^+^ T cells in Myelodysplastic Syndrome patients displayed less perforin and granzyme B secretion and higher CD95 expression. Leuk. Res..

[23] Sun J., Yu N., Li X., Wang L., Pan Y., Li X., Tao J., Chen Z., Wang G. (2016). Aberrant GITR expression on different T cell subsets and the regulation by glucocorticoid in systemic lupus erythematosus. Int. J. Rheum. Dis..

[24] Slebioda T.J., Rowley T.F., Ferdinand J.R., Willoughby J.E., Buchan S.L., Taraban V.Y., Al-Shamkhani A. (2011). Triggering of TNFRSF25 promotes CD8^+^ T-cell responses and anti-tumor immunity. Eur. J. Immunol..

[25] Benwell C.J., Taylor J., Robinson S.D. (2021). Endothelial neuropilin-2 influences angiogenesis by regulating actin pattern development and α5-integrin-p-FAK complex recruitment to assembling adhesion sites. FASEB J..

[26] Turajlic S., Xu H., Litchfield K., Rowan A., Horswell S., Chambers T., O’Brien T., Lopez J.I., Watkins T.B.K., Nicol D. (2018). Deterministic Evolutionary Trajectories Influence Primary Tumor Growth: TRACERx Renal. Cell.

[27] Fridman W.H., Zitvogel L., Sautès-Fridman C., Kroemer G. (2017). The immune contexture in cancer prognosis and treatment. Nat. Rev. Clin. Oncol..

[28] Altieri D.C. (2008). Survivin, cancer networks and pathway-directed drug discovery. Nat. Rev. Cancer.

[29] Carbonnelle-Puscian A., Copie-Bergman C., Baia M., Martin-Garcia N., Allory Y., Haioun C., Crémades A., Abd-Alsamad I., Farcet J.P., Gaulard P. (2009). The novel immunosuppressive enzyme IL4I1 is expressed by neoplastic cells of several B-cell lymphomas and by tumor-associated macrophages. Leukemia.

[30] McGranahan N., Furness A.J., Rosenthal R., Ramskov S., Lyngaa R., Saini S.K., Jamal-Hanjani M., Wilson G.A., Birkbak N.J., Hiley C.T. (2016). Clonal neoantigens elicit T cell immunoreactivity and sensitivity to immune checkpoint blockade. Science.

[31] Anderson A.C., Joller N., Kuchroo V.K. (2016). Lag-3, Tim-3, and TIGIT: Co-inhibitory Receptors with Specialized Functions in Immune Regulation. Immunity.

[32] Hakimi A.A., Reznik E., Lee C.H., Creighton C.J., Brannon A.R., Luna A., Aksoy B.A., Liu E.M., Shen R., Lee W. (2016). An Integrated Metabolic Atlas of Clear Cell Renal Cell Carcinoma. Cancer Cell.

[33] Love M.I., Huber W., Anders S. (2014). Moderated estimation of fold change and dispersion for RNA-seq data with DESeq2. Genome Biol..

[34] Chen Y., Zhou X., Xie Y., Wu J., Li T., Yu T., Pang Y., Du W. (2023). Establishment of a Seven-Gene Signature Associated with CD8^+^ T Cells through the Utilization of Both Single-Cell and Bulk RNA-Sequencing Techniques in Clear Cell Renal Cell Carcinoma. Int. J. Mol. Sci..

[35] Gu Z., Eils R., Schlesner M. (2016). Complex heatmaps reveal patterns and correlations in multidimensional genomic data. Bioinformatics.

[36] Gustavsson E.K., Zhang D., Reynolds R.H., Garcia-Ruiz S., Ryten M. (2022). ggtranscript: An R package for the visualization and interpretation of transcript isoforms using ggplot2. Bioinformatics.

[37] Sharma G., Colantuoni C., Goff L.A., Fertig E.J., Stein-O’Brien G. (2020). projectR: An R/Bioconductor package for transfer learning via PCA, NMF, correlation and clustering. Bioinformatics.

[38] Hong K., Cen K., Chen Q., Dai Y., Mai Y., Guo Y. (2023). Identification and validation of a novel senescence-related biomarker for thyroid cancer to predict the prognosis and immunotherapy. Front. Immunol..

[39] Shi Y., Wang Y., Dong H., Niu K., Zhang W., Feng K., Yang R., Zhang Y. (2023). Crosstalk of ferroptosis regulators and tumor immunity in pancreatic adenocarcinoma: Novel perspective to mRNA vaccines and personalized immunotherapy. Apoptosis.

[40] Zhao M., Zhang Q., Song Z., Lei H., Li J., Peng F., Lin S. (2023). ATP2C2 as a novel immune-related marker that defines the tumor microenvironment in triple-negative breast cancer. Transl. Cancer Res..

[41] Hänzelmann S., Castelo R., Guinney J. (2013). GSVA: Gene set variation analysis for microarray and RNA-seq data. BMC Bioinform..

[42] Liberzon A., Birger C., Thorvaldsdóttir H., Ghandi M., Mesirov J.P., Tamayo P. (2015). The Molecular Signatures Database (MSigDB) hallmark gene set collection. Cell Syst..

[43] Wang T., Dai L., Shen S., Yang Y., Yang M., Yang X., Qiu Y., Wang W. (2022). Comprehensive Molecular Analyses of a Macrophage-Related Gene Signature with Regard to Prognosis, Immune Features, and Biomarkers for Immunotherapy in Hepatocellular Carcinoma Based on WGCNA and the LASSO Algorithm. Front. Immunol..

[44] Li X., Lei J., Shi Y., Peng Z., Gong M., Shu X. (2024). Developing a RiskScore Model based on Angiogenesis-related lncRNAs for Colon Adenocarcinoma Prognostic Prediction. Curr. Med. Chem..

[45] Tastsoglou S., Skoufos G., Miliotis M., Karagkouni D., Koutsoukos I., Karavangeli A., Kardaras F.S., Hatzigeorgiou A.G. (2023). DIANA-miRPath v4.0: Expanding target-based miRNA functional analysis in cell-type and tissue contexts. Nucleic Acids Res..

[46] Jardim D.L., Goodman A., de Melo Gagliato D., Kurzrock R. (2021). The Challenges of Tumor Mutational Burden as an Immunotherapy Biomarker. Cancer Cell.

[47] Zhou H., Sun D., Miao C., Tao J., Ge C., Chen T., Li H., Hou H. (2023). The stage-dependent prognostic role of ARID1A in hepatocellular carcinoma. Transl. Cancer Res..

[48] Zhang W., Ji L., Wang X., Zhu S., Luo J., Zhang Y., Tong Y., Feng F., Kang Y., Bi Q. (2021). Nomogram Predicts Risk and Prognostic Factors for Bone Metastasis of Pancreatic Cancer: A Population-Based Analysis. Front. Endocrinol..

[49] He Z., Gu Y., Yang H., Fu Q., Zhao M., Xie Y., Liu Y., Du W. (2023). Identification and verification of a novel anoikis-related gene signature with prognostic significance in clear cell renal cell carcinoma. J. Cancer Res. Clin. Oncol..

